# Improving postural stability through proprioceptive training in dogs

**DOI:** 10.3389/fvets.2025.1645875

**Published:** 2025-07-23

**Authors:** Christiane Lutonsky, Nadja Affenzeller, Masoud Aghapour, Julia Wegscheider, Christian Peham, Alexander Tichy, Barbara Bockstahler

**Affiliations:** ^1^Section of Physical Therapy and Rehabilitation, Small Animals Surgery, Clinical Centre for Small Animal Health and Research, Clinical Department for Small Animals and Horses, University of Veterinary Medicine, Vienna, Austria; ^2^Behavioural Medicine, Clinical Centre for Small Animal Health and Research, Clinical Department for Small Animals and Horses, University of Veterinary Medicine, Vienna, Austria; ^3^Movement Science Group, Clinical Centre for Equine Health and Research, Clinical Department for Small Animals and Horses, University of Veterinary Medicine, Vienna, Austria; ^4^Department of Biological Sciences and Pathobiology, Platform for Bioinformatics and Biostatistics, Centre of Biological Sciences, University of Veterinary Medicine, Vienna, Austria

**Keywords:** posturography, proprioceptive training, center of pressure, canine rehabilitation, postural stability

## Abstract

Postural stability (PS) is essential for functional mobility and rehabilitation. While posturography and center of pressure (COP) parameters are commonly used to assess PS, little is known about the effects of proprioceptive training programs in dogs. This study aimed to evaluate the effects of a 4-week training program in dogs on a motorized proprioceptive training platform which creates a curved movement in the 3 planes of space that follows Elispheric® trajectories (Imoove-vet®, Allcare Innovations, France). Twenty dogs were divided into a training group and a control group. Five conventional COP parameters were measured under 4 different conditions (neutral, uphill, downhill, perturbed standing) pre- and post-intervention. The primary outcomes included statistically significant improvements in craniocaudal (CCD%), mediolateral (MLD%) displacement, and support surface (SS%). Dogs participating in the training group showed statistically significant reductions in COP excursions post-intervention, specifically CCD% during perturbed standing, MLD% during downhill standing, and SS% during uphill standing. Compared to the control group, the training group showed a statistically significant reduction in CCD% and SS% during uphill standing, and MLD% during downhill standing post-intervention. No statistically significant changes were observed, and effect sizes remained below Cohen’s *d* < 0.5 in the control group. In contrast, large training effects (*d* > 0.8) for all significantly decreased parameters were found in the training group. The results support the effectiveness of proprioceptive training in improving PS specifically under biomechanically challenging conditions highlighting the relevance of including these tasks in PS assessment and training protocols. Further research is warranted in orthopedically and neurologically diseased populations to explore therapeutic applications.

## 1 Introduction

Effective exercise and rehabilitation programs are crucial for optimizing canine physical health, performance, and well-being. Whether for working dogs, canine athletes, or companion animals, structured exercise plays a key role in enhancing strength and coordination (1). One aspect of exercise that is becoming increasingly important is postural stability (PS), which underpins a dog’s ability to maintain balance and safely navigate in challenging environments. Therefore PS is essential for efficient movement, injury prevention, and overall quality of life (2–5).

PS relies on a complex interplay between the central and peripheral nervous systems, musculoskeletal structures, and sensory inputs (6–8). In both human and veterinary research, PS is widely recognized as a critical component of mobility, the ability to maintain posture and perform controlled movements across different positions and environments, and functional independence (7–10). While substantial progress has been made in understanding PS mechanisms in humans, research on PS in animals, particularly dogs, remains comparatively limited. With the increasing interest in canine rehabilitation, sports medicine, and age-related mobility limitations (2, 3, 5, 11), there is a need for proprioceptive training protocols specifically targeting impairments in PS in dogs.

In dogs, PS assessments are frequently conducted using force or pressure plate analysis. These assessments provide objective data on stability by evaluating the body’s center of pressure (COP). The COP moves within the functional base of support (BOS), which is the area that reflects the limits of stability (12). If the COP exceeds the functional BOS, a protective step is typically initiated to prevent falling (4, 12). The most frequently used COP parameters are the displacement in craniocaudal (CLD) and mediolateral (MLD) directions within the functional BOS, the average speed (AS), the support surface (SS) and the statokinesiogram length (total trajectory length of the COP, L). These parameters provide critical insights into an animal’s postural efficiency and performance, with deviations from normal patterns indicative of impaired PS (3, 11, 13–15). Generally, smaller COP displacements and reduced AS, SS and L are associated with superior PS (3, 16).

It has already been shown that rehabilitation programs including proprioceptive training designed to improve PS significantly benefit human patients (17). Proprioceptive training interventions increase the BOS, reduce postural sway, and enhance neuromuscular coordination (18). In humans, these programs typically incorporate exercises that narrow the BOS, challenge proprioceptive input, and introduce controlled perturbations to stimulate adaptive postural responses (19–21). The movement of the COP within the BOS reflects the underlying muscle activity required to maintain an upright posture (3, 4, 22).

In dogs, aging, orthopedic conditions, and neurological disorders often impair PS (3, 11, 13, 14). Healthy senior dogs display greater postural sway and reduced stability, likely due to diminished neuromuscular control and musculoskeletal degeneration when compared to young dogs (3, 11). Dogs with osteoarthritis or joint pain also show compromised stability, as indicated by increased COP displacement (13, 14). These findings highlight the importance of targeted rehabilitation strategies in dogs to reduce PS impairments and support functional mobility in dogs.

Veterinary researchers have explored how dogs and horses adapt their posture under various conditions, including loss of vision when blindfolded (3, 23, 24), the use of proprioceptive balance pads (25), and exposure to external perturbations (4, 26). Exercise-based therapeutic training protocols have been shown to improve static body weight distribution, reduce pain-related functional disability, and enhance stifle joint function in dogs with stifle injuries (27). In horses, rehabilitation exercises that included proprioceptive exercises significantly reduced COP parameters, especially under challenging conditions like standing on a proprioceptive balance pad (25). Further, equine research found a significant effect of underwater treadmill training on COP parameters when compared to a land treadmill (24).

Despite the growing application of proprioceptive exercises in canine rehabilitation and physiotherapy—such as standing on balance boards—standardized and validated study protocols are still lacking. This limits the ability to systematically evaluate their effectiveness and to adjust exercise interventions targeting PS accordingly. Recent studies have started to address this gap. One investigation used a motorized training platform which creates a curved movement in the 3 planes of space that follows Elispheric® trajectories. Different combinations of speed and amplitude (inclination of the platform) showed that the amplitude, rather than the speed, had a greater effect on COP parameters, indicating a stronger challenge to PS (4). These findings suggest that motorized training platforms might be an applicable tool to tailor exercise parameters and assess treatment outcome in a more standardized way.

Pressure-sensitive proprioceptive platforms produce highly reliable measurements in healthy dogs, with consistent COP data—such as L, SS, and average velocity—across multiple time points. Perturbations revealed moderate training effects, implying that repeated exposure may influence performance (26). However, no studies have yet evaluated the impact of a proprioceptive training program on COP parameters in dogs.

The present study aims to address this gap by evaluating the effects of a 4-week proprioceptive training program on PS. By assessing pre- and post-intervention COP parameters using a pressure measurement plate, this study seeks to provide empirical evidence on the effect of a proprioceptive training intervention in dogs. PS development was assessed under 4 different conditions: neutral (standing on even ground), standing uphill, standing downhill and perturbed standing on a motorized training platform. The hypotheses were that standing uphill leads to smaller changes in PS parameters than standing downhill and that external perturbations cause the largest PS deviations. Further, we hypothesized that PS would improve after completion of the training program, which is reflected by a decrease in COP parameters, especially in the 3 aggravated conditions.

## 2 Materials and methods

### 2.1 Approval and consent

This study was approved by the Ethics and Animal Welfare Committee of the University of Veterinary Medicine, Vienna, in accordance with the University’s guidelines for Good Scientific Practice (ETK-148/10/2021 and ETK-168/10/2022). Informed consent was obtained from all dog caretakers.

### 2.2 Animals and inclusion criteria

This was a prospective, randomized, controlled intervention study conducted over a period of 6 weeks including 20 client-owned dogs. The criteria for inclusion required the dogs to be free from any diagnoses or clinical signs of orthopedic, neurological, or visual disorders, with an age range of 1–8 years and a body weight between 15 and 35 kg. Body height (measured from the ground level to margo dorsalis of the scapula) and body length (measured from the greater tubercle of the humerus to the ischial tuberosity) were recorded. Each dog underwent a comprehensive clinical evaluation by a veterinarian, which included visual gait observation, orthopedic and neurological assessments, and an objective gait analysis.

For the gait analysis, a pressure measurement plate measuring 203 × 54.2 cm (FDM Type 2, Zebris Medical GmbH, Allgäu, Germany) equipped with 15,360 sensors and a measuring frequency of 100 Hz was utilized. The plate was covered with a 1-mm thick black polyvinyl chloride rubber mat to prevent slipping. Initially, the dogs were allowed to explore the measurement area freely to become accustomed to the environment. Data collection was conducted during both walking and trotting, ensuring that at least 5 valid trials were recorded for each gait. A trial was considered valid if the dog moved across the plate at a consistent pace without altering its gait, turning its head, pulling on the leash, or interacting with the owner. Speed variations while crossing the plate were limited to ±0.3 m/s, and acceleration differences to ±0.5 m/s^2^ (28–30). For inclusion, the symmetry index (SI) of peak vertical force (PFz) and vertical impulse (IFz) had to be below 3%, which is the margin typically used to differentiate between a symmetric and asymmetric gait (31).

### 2.3 Study protocol

#### 2.3.1 Measurement conditions

Static measurements were conducted under 4 conditions: standing on level ground (neutral), standing downhill and uphill on a 20° slope and standing on a motorized training platform (Imoove-vet® platform, Allcare Innovations, France) (perturbated). The order of the four measurement conditions – (1) neutral stance, (2) downhill, (3) uphill, and (4) perturbed stance – was randomized. The randomization was performed using the Excel function “RAND” to assign a random sequence for each dog. Only whole numbers between 1 and 4 were used to ensure valid condition assignments.

**Figure 1:**
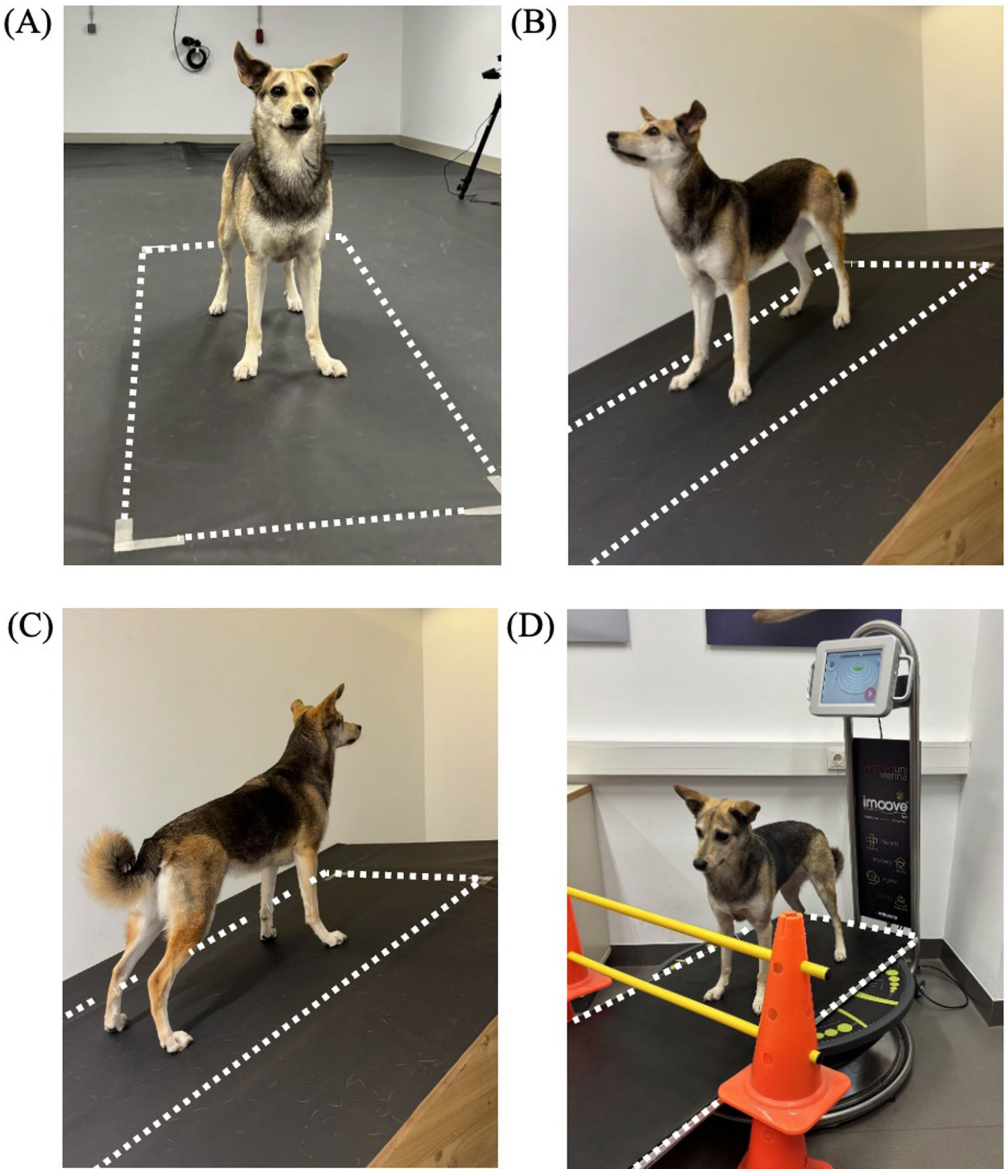
Measurement setup for each condition, including neutral standing **(A)**, downhill standing **(B)**, uphill standing **(C)** and perturbed standing on a motorized training platform (Imoove-vet® platform, Allcare Innovations, France) **(D)**. The pressure measurement plate is indicated with dotted lines.

For each condition, data were collected using a pressure measurement plate. The same plate as for the gait analysis was used for the neutral condition, while a smaller sized (149 × 54.2 cm) pressure measurement plate with the same features as described above (FDM-1.5, Zebris Medical GmbH, Allgäu, Germany) was utilized for the sloped and perturbation condition. The pressure plate for these conditions was either integrated into the slope setup or positioned on top of the motorized training platform. Since the plate was longer than the training platform, two cavaletti were used to ensure that the dogs did not pass over the edge. In all conditions the measurement plate was covered with a black, 1-mm-thick rubber mat made of polyvinyl chloride (Figure 1).

The caregiver guided the dog on top of the pressure plate into a straight and square posture by using positive reinforcement methods. The dogs had to stand still on the pressure measurement plate with all 4 paws placed on the plate and with a straight and symmetrical limb position, without any movement of the body, head, tail, limbs, or paws. For this purpose, the owner stood in front of the animal to maintain its attention during the measurement procedure. After each measurement, the animal was rewarded with a treat and was allowed to rest. The required measurement duration was set to 5 s of quiet standing. As recommended, 2 valid passes of 5 s measurements per condition were collected and analyzed for each dog (32).

The setting selected in this study was previously used in postural research in healthy dogs and consisted of an amplitude of 30% or ± 2.4° angle and a speed of 10% or 6 rpm (4, 33). This setting ensured a maximum challenge for PS that was considered safe for all dogs, without the need for protective steps (4). Each measurement was filmed with a Panasonic camera (model NV-MX500) to ensure no head, limb, and tail movements during data analysis.

#### 2.3.2 Proprioceptive training

After the baseline measurements (T0) in all 4 conditions, the dogs were randomly assigned to group C (control group) or group T (training group). The randomization was performed using the Excel function “RAND,” followed by ranking to generate a randomized sequence of whole numbers (1 or 2) for group allocation. Group T participated in a 4-week training program consisting of 2 proprioceptive training sessions per week. Each training comprised a 10-min session, where the dog had to maintain a standing position while the platform was rotating at the setting previously described. If the dog sat down, it was motivated to resume standing by using positive reinforcement methods such as treats. To further support the dog, the standing posture was intermittently rewarded throughout the session based on the individual’s needs. The demographic data of both groups can be found in Table 1.

**Table 1:** Demographics of the participating dogs in group C (control) and group T (training), including mean ± SD of body mass, height and length and *p*-values of the comparison between groups.

Group	*N*	Age (m)	Body mass (kg)	Height (cm)	Length (cm)
C	10	48.70 ± 15.05	21.16 ± 6.70	54.25 ± 5.27	58.10 ± 6.74
T	10	53.50 ± 24.07	22.70 ± 5.24	56.25 ± 6.06	55.75 ± 4.32
*p*-value		0.599	0.574	0.441	0.366

Five weeks after the baseline assessment, both groups were re-evaluated in all 4 conditions. By this time, group T had completed the 4-week training on the motorized training platform, including a 1 week break before the second measurements (T1). Caregivers were instructed not to make any changes to their dogs’ usual level of physical activity throughout the study period.

#### 2.3.3 Data analysis

All parameters were analyzed using a custom software Pressure Analyzer (Michael Schwanda, version 4.9.3.0), which was then exported to Microsoft Excel 2016.

The following parameters were used for the evaluation of the inclusion criteria during objective gait analysis:

- Mean speed (m/s) and acceleration (m/s^2^) were calculated for the left forelimb.
- Symmetry index (SI) expressed as a percentage (SI%), was calculated for both parameters (PFz and IFz) according to the following modified equation (34):

$$\text{SIXFz}\;(\%)=\mathrm{abs}(\frac{\left[\text{XFzLLx}–\text{XFzRLx}\right]}{\left[\text{XFzLLx}+\text{XFzRLx}\right]})*100$$

Where XFz is the mean value of peak vertical force (PFz) or vertical impulse (IFz) of valid steps, LLx is the left front or hindlimb, and RLx is the right front or hind; Perfect symmetry between the right and left front or hindlimbs was assigned a value of 0%.

During the posturographic analysis, all data were low-pass filtered using a 4^th^-order Butterworth Filter with a cutoff frequency of 10 Hz (35). The following parameters were analyzed:

##### 2.3.3.1 Base of support

- BOS (cm^2^): area enclosed by the coordinates of the center of the paws.
- Base of support length (BOS L, cm): distance between the paw center of the front and hindlimbs.
- Base of support width (BOS W, cm): distance between the paw center of the left and right limbs.

##### 2.3.3.2 Center of pressure

- Craniocaudal displacement (mm): Deviation on the craniocaudal axis. It was normalized to the BOS L and expressed as a percentage (CCD%).
- Mediolateral displacement (mm): Deviation on the lateral axis. It was normalized to the BOS W and expressed as a percentage (MLD%).
- Statokinesiogram length (m): the length of the line that joins the points of the COP trajectory. It was normalized to the BOS and expressed as a percentage (L%).
- Support surface or statokinesiogram (mm^2^): The area determined by an ellipse that contains 90% of the points of the COP trajectory. It was normalized to the BOS and expressed as a percentage (SS%).
- Mean speed (AS) (mm/s) of COP sway.

##### 2.3.3.3 Percentage difference

The percentage difference between T0 and T1 in all 4 conditions was calculated for each dog. Individual percentage COP reactions were expressed as ΔCCD%, ΔMLD%, ΔL%, ΔAS, ΔSS% using the following formula exemplarily shown for ΔCCD%:


$$\text{ΔCCD}\%=\frac{\mathrm{CCD}\%(T1)-\mathrm{CCD}\%(T0)}{\mathrm{CCD}\%(T0)}*100$$

### 2.4 Statistical analysis

All statistical analyses were performed using IBM SPSS v29. To evaluate potential differences between groups, independent two-tailed t-tests were performed for body height, body length, age, and body mass.

To analyze the effects of different measurement conditions, data from all dogs at baseline (T0) were pooled and assessed using linear mixed-effects models, with conditions included as fixed factors. Subsequently, the dogs were divided into two groups (Group C and Group T) and separate analyzes were conducted to compare the groups at T0 and T1, as well as to investigate the effect of the training program over time within group T. Sidak’s alpha correction was applied to adjust for multiple comparisons. The assumption of normality was tested using the Shapiro–Wilk test. Statistical significance was considered at an alpha level of *p* < 0.05.

To quantify the magnitude of an observed difference between groups, Cohen’s *d* was calculated for all COP parameters. Effect sizes were categorized as negligible (<0.2), small (0.2–0.5), moderate (0.5–0.8), and large (>0.8) (26).

## 3 Results

### 3.1 Comparison of demographic data

No significant differences between groups were found for age, body mass, body height, body length (Table 1).

### 3.2 Base of support

Descriptive statistics of the baseline measurements of all dogs are displayed in the Supplementary Table S1. No significant differences were found during baseline measurements of all dogs in BOS parameters between conditions (Supplementary Table S2). Looking at the groups separately, mean for each group and each measurement condition can be found in Supplementary Table S3. No significant differences were found between measurement timepoints T0 and T1 (Supplementary Table S4) and groups C and T (Supplementary Table S5).

### 3.3 Center of pressure

#### 3.3.1 Effect of measurement conditions

When analyzing the baseline data (T0) of all 20 dogs, the perturbed standing condition resulted in significantly higher values in all 5 investigated COP parameters when compared to the other 3 conditions (Tables 2, 3). In addition, compared to the neutral condition, both standing uphill and standing downhill led to a significant increase in CCD% and SS%. Moreover, standing downhill was associated with a significantly greater MLD% when compared to the neutral condition. No significant differences in all COP parameters were observed between standing uphill and standing downhill. For a graphical illustration see Supplementary Figure S1.

**Table 2:** Mean values ± standard deviation of the craniocaudal (CCD%) and mediolateral (MLD%) center of pressure displacement, the statokinesiogram length (L%), the support surface (SS%) and average speed (AS) of the measurement conditions across all subjects during the measurement timepoint baseline (T0).

Condition	CCD%	MLD%	L%	SS%	AS (mm/s)
Neutral	1.21 ± 0.25^1,2,3^	1.32 ± 0.30^1,3^	0.10 ± 0.03^3^	0.09 ± 0.03^1,2,3^	20.75 ± 7.00^3^
Downhill	1.63 ± 0.48^*,3^	1.64 ± 0.30^*,3^	0.13 ± 0.04^3^	0.15 ± 0.07^*,3^	24.24 ± 7.62^3^
Uphill	1.54 ± 0.32^*,3^	1.63 ± 0.55^3^	0.12 ± 0.04^3^	0.17 ± 0.10^*,3^	21.40 ± 7.51^3^
Perturbated	9.86 ± 2.04^*,1,2^	26.83 ± 6.82^*,1,2^	0.45 ± 0.21^*,1,2^	31.88 ± 13.42^*,1,2^	39.39 ± 8.97^*,1,2^

^*^Significant difference to neutral condition; ^1^significant difference to downhill; ^2^significant difference to uphill; ^3^significant difference to perturbed.

**Table 3:** *p*-values of the comparison between all 4 measurement conditions of all dogs during baseline measurements (T0), including the craniocaudal (CCD%) and mediolateral (MLD%) center of pressure displacement, the statokinesiogram length (L%), the support surface (SS%) and average speed (AS).

Condition I	Condition II	CCD%	MLD%	L%	SS%	AS (mm/s)
Neutral	Downhill	**0.010**	**0.009**	0.091	**0.005**	0.596
	Uphill	**0.005**	0.177	0.877	**0.024**	1.000
	Perturbed	**<0.001**	**<0.001**	**<0.001**	**<0.001**	**<0.001**
Downhill	Neutral	**0.010**	**0.009**	0.091	**0.005**	0.596
	Uphill	0.984	1.000	0.763	0.994	0.812
	Perturbed	**<0.001**	**<0.001**	**<0.001**	**<0.001**	**<0.001**
Uphill	Neutral	**0.005**	0.177	0.877	**0.024**	1.000
	Downhill	0.984	1.000	0.763	0.994	0.812
	Perturbed	**<0.001**	**<0.001**	**<0.001**	**<0.001**	**<0.001**
Perturbed	Neutral	**<0.001**	**<0.001**	**<0.001**	**<0.001**	**<0.001**
	Downhill	**<0.001**	**<0.001**	**<0.001**	**<0.001**	**<0.001**
	Uphill	**<0.001**	**<0.001**	**<0.001**	**<0.001**	**<0.001**

Numbers in bold represent *p* < 0.05.

#### 3.3.2 Effect of the training program

At baseline (T0), no significant differences were found between groups C and T in any of the 5 COP parameters (Tables 4, 5). Furthermore, no significant differences between T0 and T1 were observed in group C in any COP parameters (Table 6). Based on calculated Cohen’s d, a small effect was observed for L%, SS%, and AS during neutral standing (*d* = 0.45, 0.38, and 0.38, respectively); for L% during downhill standing (*d* = 0.24); for CCD% and AS% during uphill standing (*d* = 0.40 and 0.23, respectively); and for CCD% and AS% during perturbed standing (*d* = 0.27 and 0.43, respectively). All remaining 12 calculated effect sizes within the control group remained negligible (*d* < 0.2) (Table 7).

**Table 4:** Descriptive analysis of all 4 conditions of both control group (C) and training group (T) at both measurement timepoints (baseline T0 and 5 weeks later T1).

Condition	Day	Group	CCD%	MLD%	L%	SS%	AS (mm/s)
Neutral	T0	C	1.22 ± 0.26	1.27 ± 0.24	0.11 ± 0.04	0.09 ± 0.03	21.84 ± 6.97
		T	1.20 ± 0.26	1.36 ± 0.36	0.10 ± 0.02	0.09 ± 0.04	19.65 ± 7.23
	T1	C	1.21 ± 0.40	1.31 ± 0.42	0.13 ± 0.07	0.11 ± 0.07	25.95 ± 13.70
		T	0.97 ± 0.15	1.16 ± 0.23	0.09 ± 0.03	0.06 ± 0.02	19.35 ± 7.55
Downhill	T0	C	1.70 ± 0.56	1.61 ± 0.32	0.14 ± 0.05	0.16 ± 0.08	24.17 ± 6.82
		T	1.56 ± 0.40	1.67 ± 0.29*	0.13 ± 0.03	0.14 ± 0.04	24.31 ± 8.72
	T1	C	1.61 ± 0.59	1.66 ± 0.43^#^	0.15 ± 0.08	0.15 ± 0.10	24.20 ± 10.11
		T	1.34 ± 0.42	1.32 ± 0.21^*,#^	0.14 ± 0.05	0.10 ± 0.03	23.82 ± 7.39
Uphill	T0	C	1.52 ± 0.27	1.57 ± 0.67	0.13 ± 0.05	0.15 ± 0.11	23.86 ± 7.22
		T	1.56 ± 0.38	1.69 ± 0.42	0.11 ± 0.04	0.18 ± 0.10^*^	18.94 ± 7.32
	T1	C	1.67 ± 0.44^#^	1.48 ± 0.35	0.12 ± 0.06	0.15 ± 0.05^#^	21.86 ± 9.59
		T	1.29 ± 0.23^#^	1.32 ± 0.26	0.10 ± 0.02	0.10 ± 0.03^*,#^	20.10 ± 6.37
Perturbed	T0	C	9.46 ± 2.18	24.52 ± 6.23	0.46 ± 0.24	28.38 ± 12.47	37.82 ± 7.01
		T	10.27 ± 1.91^*^	29.13 ± 6.90	0.44 ± 0.19	35.38 ± 14.05	40.96 ± 10.73
	T1	C	8.87 ± 2.18	23.36 ± 7.09	0.47 ± 0.16	25.93 ± 14.93	41.22 ± 8.62
		T	8.10 ± 1.04^*^	25.44 ± 8.25	0.44 ± 0.12	25.80 ± 10.94	39.10 ± 6.45

Mean values and standard deviation of the craniocaudal (CCD%) and mediolateral center of pressure displacement (MLD%), the statokinesiogram length (L%), the support surface (SS%) and average speed (AS) are shown. ^*^Significant difference between measurement time points; ^#^significant difference between groups.

**Table 5:** Comparison of *p*-values between control group C and training group T of all center of pressure (COP) parameters, including the craniocaudal (CCD%) and mediolateral COP displacement (MLD%), the statokinesiogram length (L%), the support surface (SS%) and average speed (AS) for both measurement timepoints baseline (T0) and 5 weeks later (T1).

Day	Condition	CCD%	MLD%	L%	SS%	AS (mm/s)
T0	Neutral	0.853	0.511	0.434	0.749	0.499
	Downhill	0.548	0.648	0.683	0.401	0.969
	Uphill	0.806	0.615	0.309	0.591	0.148
	Perturbed	0.388	0.134	0.828	0.254	0.449
T1	Neutral	0.098	0.330	0.098	0.058	0.199
	Downhill	0.257	**0.041**	0.592	0.131	0.924
	Uphill	**0.026**	0.271	0.380	**0.028**	0.634
	Perturbed	0.328	0.552	0.681	0.982	0.543

Numbers in bold represent *p* < 0.05.

**Table 6:** Comparison of *p*-values within control group C and training group T of all center of pressure (COP) parameters, including the craniocaudal (CCD%) and mediolateral COP displacement (MLD%), the statokinesiogram length (L%), the support surface (SS%) and average speed (AS) for both measurement timepoints baseline (T0) and 5 weeks later (T1).

Group	Condition	CCD%	MLD%	L%	SS%	AS (mm/s)
C	Neutral	0.956	0.789	0.214	0.321	0.331
	Downhill	0.697	0.738	0.524	0.727	0.992
	Uphill	0.346	0.656	0.723	0.849	0.567
	Perturbed	0.488	0.718	0.892	0.680	0.371
T	Neutral	0.088	0.165	0.793	0.121	0.943
	Downhill	0.328	**0.021**	0.749	0.257	0.898
	Uphill	0.084	0.073	0.800	**0.038**	0.739
	Perturbed	**0.015**	0.257	0.941	0.113	0.623

Numbers in bold represent *p* < 0.05.

**Table 7:** Effect sizes (Cohen’s *d*) for each center of pressure parameter across the four tested conditions (neutral, uphill, downhill, perturbed) comparing baseline (T0) and post-intervention (T1) values within groups.

Condition	Group	CCD%	MLD%	L%	SS%	AS (mm/s)
Neutral	C	0.02	0.11	0.45	0.38	0.38
	T	1.05	0.68	0.19	0.95	0.04
Downhill	C	0.15	0.13	0.24	0.12	0.00
	T	0.54	1.37	0.18	0.93	0.06
Uphill	C	0.40	0.17	0.13	0.08	0.23
	T	0.86	1.08	0.16	1.03	0.17
Perturbed	C	0.27	0.17	0.05	0.18	0.43
	T	1.41	0.49	0.04	0.76	0.21

Cohen’s *d* was calculated to assess the magnitude of change between time points. Effect sizes were interpreted as follows: negligible (<0.2), slight (0.2–0.5), moderate (0.5–0.8), and clear (>0.8) (26). Values are presented separately for the training group (group T) and the control group (group C).

Following the 4-week proprioceptive training program, Group T showed the following significantly lower COP values when compared to Group C: CCD% and SS% during uphill standing, and MLD% during downhill standing (Table 5).

Within Group T, the proprioceptive training program also led to significant decreases in CCD% during perturbed standing, MLD% during downhill standing, and SS% during uphill standing (Table 6). All statistically significant changes were accompanied by large effect size as indicated by Cohen’s *d* > 0.8. Further clear effects (*d* > 0.8) were observed in Group T for CCD% during neutral and uphill standing, MLD% during uphill standing, and SS% during neutral and downhill standing (Table 7).

**Figure 2:**
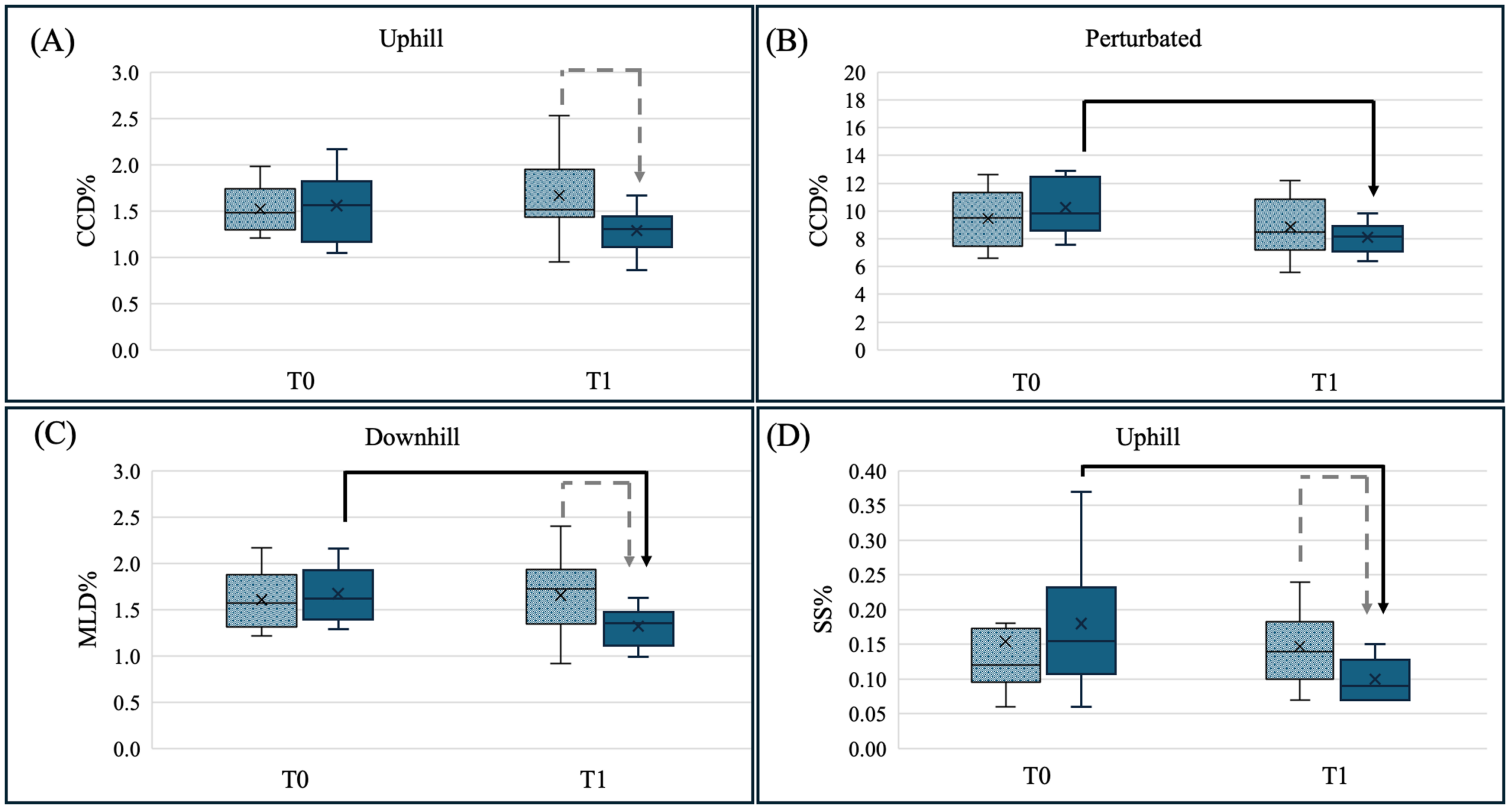
Illustration of the significant results of the pairwise comparison between control group C (dotted-grey column) and training group T (blue, solid column) for both measurement timepoints baseline (T0) and 5 weeks later (T1) for the conditions: **(A)** craniocaudal center of pressure (COP) displacement (CCD%) uphill standing, **(B)** craniocaudal COP displacement (CCD%) perturbed standing, **(C)** mediolateral COP displacement (MLD%) for downhill standing and **(D)** support surface (SS%) for uphill standing. Significant differences between T0 and T1 are marked with a black arrow and significant differences between groups C and T are marked with a grey dotted arrow (*p* < 0.05). These plots display the median as the measure of central tendency, along with the interquartile range (IQR) to represent the spread of the data. Whiskers indicate the range excluding outliers, and individual data points outside 1.5 × IQR are displayed as outliers.

No significant training effects were found for L% and AS in any of the tested conditions. Consistently, the calculated effect sizes for L% and SS% ranged from negligible to small (Table 7). A graphical illustration of all comparisons is provided in Supplementary Figure S2, whereas all significant differences are illustrated in Figure 2.

#### 3.3.3 Percentage change

Group T exhibited significant decreases in ΔCCD% in the perturbed standing condition and in ΔMLD% when standing downhill at T1 when compared to Group C. No significant differences between the groups were observed in the remaining conditions and COP parameters (Table 8).

**Table 8:** Mean values ± standard deviation and *p*-values of the inter-group comparison of the percentage change of craniocaudal (CCD%) and mediolateral (MLD%) center of pressure displacement, the statokinesiogram length (L%), the support surface (SS%) and average speed (AS) of the measurement conditions.

Condition	Group	ΔCCD%	ΔMLD%	ΔL%	ΔSS%	ΔAS
Neutral	C	2.79 ± 25.53	6.78 ± 27.65	25.20 ± 48.31	30.30 ± 61.93	25.20 ± 48.31
	T	−12.09 ± 18.99	−8.22 ± 26.57	4.35 ± 31.63	−13.26 ± 40.68	4.35 ± 31.63
	*p*-value	0.156	0.232	0.269	0.079	0.269
Downhill	C	−3.21 ± 16.84	−3.39 ± 16.07	6.31 ± 38.12	−12.41 ± 24.23	5.74 ± 37.81
	T	−10.35 ± 17.49	−22.18 ± 19.71	7.33 ± 40.41	−25.74 ± 21.10	7.33 ± 40.41
	*p*-value	0.364	**0.031**	0.954	0.206	0.928
Uphill	C	6.90 ± 19.46	7.30 ± 33.95	−0.25 ± 37.72	15.21 ± 28.65	−0.25 ± 37.72
	T	−5.71 ± 29.30	−8.80 ± 33.60	22.35 ± 60.28	−11.74 ± 56.28	22.35 ± 60.28
	*p*-value	0.272	0.301	0.328	0.194	0.328
Perturbed	C	−2.32 ± 16.75	−4.88 ± 11.74	13.60 ± 31.60	−4.10 ± 38.25	11.61 ± 28.51
	T	−19.36 ± 12.98	−11.33 ± 22.73	10.70 ± 38.43	−18.23 ± 41.11	1.28 ± 29.84
	*p*-value	**0.020**	0.435	0.856	0.437	0.439

Numbers in bold represent *p* < 0.05.

## 4 Discussion

This study aimed to investigate the effect of a 4-week proprioceptive training program on PS in dogs, particularly under biomechanically challenging conditions such as standing uphill, standing downhill, and perturbed standing. COP parameters were used to quantify effects on canine PS. A threefold hypothesis was formulated: first, that uphill standing would result in smaller deviations in COP parameters compared to downhill standing; second, that perturbed standing during external perturbations cause the largest COP deviations; and third, that the proprioceptive training program would improve PS under aggravated conditions, reflected by a reduction in COP excursions. The results support these hypotheses, revealing condition-dependent variations in postural mechanisms and indicating a beneficial effect of proprioceptive training on PS in young healthy dogs.

### 4.1 Effect of measurement conditions

As previously reported in human studies (9), standing downhill on a 20° slope resulted in significant changes of PS compared to standing in a neutral position. Notably, in humans this effect was found in L% and AS, whereas in our study CCD%, SS%, and MLD% were significantly affected. Further, standing uphill showed no significant effect on COP parameters in humans (9, 36) whereas dogs exhibited significant increases CCD% and SS% when standing uphill compared to neutral standing.

These differences may be partly due to measurement techniques, concerning measurement duration and repetitions, but also reflect anatomical and neuromuscular variations between both species. Dogs have a considerably greater ROM in their carpal and tarsal joints than humans in their ankle joint (37–39). This limited ROM in humans may increase passive joint stability, e.g., through tendons, reducing the demand on neuromuscular control. In contrast, the larger ROM in canine distal joints may require more active stabilization under dynamic and unstable standing conditions.

Further species-specific differences arise in the musculotendinous architecture of the lower limb. In humans, the triceps surae complex (gastrocnemius and soleus) acts via the Achilles tendon to stabilize the ankle. The soleus plays a key role in postural regulation (40). In dogs, which lack a soleus muscle, the common calcaneal tendon (tendo calcaneus communis) consists of several muscle contributions (41), potentially influencing both stability and energy storage. These structural and functional differences between species highlight the importance of considering both joint architecture and neuromuscular organization when interpreting PS and control mechanisms across quadrupeds and bipeds. Given the current paucity of comparative studies (42), drawing definitive conclusions about different mechanisms of PS between humans and animals remain challenging.

Compared to standing in a neutral position, standing downhill resulted in significant increases in 3 out of 5 COP parameters in dogs (CCD%, SS%, MLD%), while standing uphill only affected CCD% and SS%. Looking at these results, it can be argued that standing downhill resulted in a greater challenge for PS compared to standing uphill. Given that mediolateral stability is largely attributed to hip mechanisms in humans (43), in dogs the bony connection of the pelvis to the hindlimb may allow for more effective mediolateral control when compared to the soft tissue-only connection of the forelimb via synsarcosis (41). Therefore, increased hindlimb loading when standing uphill may be better compensated than forelimb loading when standing downhill.

As expected, external perturbations induced by the motorized training platform resulted in a significant increase in all COP parameters compared to all other standing conditions, confirming its value in challenging PS mechanisms. This contrasts with previous studies in which the same training platform did not significantly increase AS or L compared to a neutral stance (4). Methodological differences likely account for this discrepancy, including the duration and number of measurements, data filtering, and normalization to the BOS. This underlines the importance of consistent methodology to ensure study comparability (32).

### 4.2 Effect of the training program

No significant differences were found between time points T0 and T1 in the neutral standing condition in both groups C and T. This highlights the need to incorporate biomechanically challenging conditions such as external perturbations or sloped surfaces when evaluating group differences or certain training effects. Similar findings were reported in a study investigating the effects of ageing on PS in dogs, where no differences were found when standing in a neutral position, but healthy senile dogs showed increased COP excursions compared to young dogs when the visual system was challenged when blindfolded (3).

No significant differences were found between time points T0 and T1 in group C across condition. Similar to the results of our group C, a recent study reported small to moderate changes in COP parameters after repeated testing (twice within 2 weeks) when Cohen’s *d* was used as a measure of effect size, although no significant differences in COP parameters were found when a linear mixed effect model was used (26). We propose that these negligible (*d* < 0.2) and small (*d* < 0.5) changes likely reflect minimal adaptation due to repeated measurements rather than clinically relevant training effects. This is supported by our finding in group T; Cohen’s *d* revealed large training effects (*d* > 0.8) for all parameters that showed a significant reduction following the 4-week proprioceptive training program. Given the absence of any moderate or large effects in group C, these effect sizes further support the relevance of the observed changes and highlight the clinical value of proprioceptive training in improving PS.

As anticipated, group T showed significant reductions in COP parameters 1-week post-intervention: CCD% during perturbed standing, MLD% during downhill standing, and SS% during uphill standing. Compared to group C, these reductions remained significant for SS% when standing uphill and MLD% when standing downhill. These findings support the expected efficacy of proprioceptive training in dogs and are in line with previous survey-based results (44) and functional tests such as the three-legged standing test (27).

Furthermore, Cohen’s *d* revealed large training effects (*d* > 0.8) in group T for all parameters that showed a significant reduction following the 4-week proprioceptive training program. Given the absence of moderate or large effects in Group C, these effect sizes further support the relevance of the observed changes and highlight the clinical value of proprioceptive training in improving PS.

However, it is important to note that COP analysis, although the most commonly used method for assessing PS in dogs (10), has not previously been applied to evaluate training effects. In contrast, equine research has long used COP parameters to assess the impact of physical exercises on PS (24, 25).

Each challenging condition revealed significant effects between groups and/or time points, with reduced COP excursions in the trained dogs. MLD% was significantly reduced in group T when standing downhill, while no such effect was found when standing uphill. Further, the proprioceptive training program resulted in a significantly greater percentage change between T0 and T1 in MLD% for group T compared to group C. No significant difference between percentage change in MLD% was found when standing uphill.

When comparing all conditions, standing downhill challenged mediolateral excursion, but standing uphill had no effect on this parameter. Therefore, we suggest that while the synsarcosis between the forelimbs and the thorax might result in a decreased stability in mediolateral direction compared to the bony connection of the hindlimbs (41), mediolateral stability can be increased using proprioceptive exercises.

This finding is of particular interest for sport dogs. Injuries of the proximal forelimb are commonly described in agility with 15.80% (45) to 30.10% (46) of injuries affecting the shoulder joint or shoulder girdle musculature. The “medial shoulder syndrome,” an instability of the synsarcosis between shoulder and thorax, represent 12.70% (75/589) of injuries reported (46). Based on the observed improvements in mediolateral stability, it is conceivable that proprioceptive exercise as applied in this study may contribute to injury prevention, particularly for proximal limb injuries in sport dogs. While this was not directly assessed in our study, and our sample did not specifically include individuals with musculoskeletal disorders, the findings suggest a potential preventive role that warrants further investigation.

The proprioceptive training program resulted in no significant effect during external perturbations in mediolateral stability but resulted in a significant decrease in CCD% between T0 and T1. Accordingly, group T showed a significantly greater percentage change from T0 to T1 compared to group C. Therefore, we propose that the proprioceptive training program used in this study resulted in a significant improvement of craniocaudal stability during perturbed standing on a motorized training platform.

Previous research suggested that mechanical perturbations represent a stronger challenge on mediolateral stability compared to craniocaudal (4). Therefore, it could be suspected that a proprioceptive training on such devices should increase mediolateral stability. However, a 4-week training program might not be enough to increase mediolateral stability in the most challenging condition used in this study. However, as previously mentioned, the comparison of results based on different measurement techniques and data analysis should be performed with caution.

The main limitation of this study is the relatively small sample size (10 dogs per group), which is common phenomenon in clinical research (3, 4). To control for a low sample size, we aimed at minimizing variability by selecting dogs of similar age and body conformation (groups controlled for weight, height and length). Since the mat only measures vertical forces, the sensitivity decreases when tilted by a factor of the direction cosine. Due to the limited number of repeated measurements, standard error of measurement was not calculated. However, the absence of change in the control group, the statistically significant differences and large training effects (*d* > 0.8) in the training group suggest that measurement error is unlikely to explain the observed effects.

Future studies should focus on the effects of PS training in orthopedically or neurologically affected animals. Based on our results, we recommend the inclusion of biomechanically challenging conditions such as standing on inclined and declined slopes and under external perturbations when assessing PS across groups or evaluating therapeutic outcomes. In addition, previous studies have suggested that blindfolded stance may further challenge postural control and could be considered in future protocols (3).

## 5 Conclusion

This study demonstrated that a 4-week proprioceptive training program improved PS in dogs, particularly under biomechanically challenging conditions such as standing uphill, standing downhill, and perturbed standing. The reductions in COP excursions in the trained group, especially when standing uphill and standing downhill, indicate improved postural control after the intervention. The findings underline the importance of including biomechanical challenges when assessing PS. This research is among the first to apply COP analysis to evaluate training effects in dogs, revealing condition-specific improvements. While standing uphill primarily challenged craniocaudal displacement, standing downhill and external perturbations impacted both axes. Despite a limited sample size, the results provide a basis for further studies, particularly in clinical populations. Incorporating inclined and declined slopes and external perturbation-based tasks into assessment protocols may enhance the detection of PS impairments and training effects in both healthy and affected animals.

## Data Availability

The original contributions presented in the study are included in the article/Supplementary material, further inquiries can be directed to the corresponding author.
