# Supplementary material for: Improving postural stability through proprioceptive training in dogs
Source: Front Vet Sci. 2025 Jul 23;12:1645875. doi: 10.3389/fvets.2025.1645875 (PMC12327393; doi:10.3389/fvets.2025.1645875)
Supplement: Supplementary file 1 [file Table_1.docx]

Supplementary Material

Table S1 Mean values ± standard deviation during the baseline measurements (T0) of all study participants of the base of support (BOS), its length (BOS L) and its width (BOS W).

| Condition | BOS (cm^2^) | BOS L (cm) | BOS W (cm) |
| --- | --- | --- | --- |
| Neutral | 1009.99 ± 161.15 | 52.74 ± 6.44 | 19.52 ± 2.41 |
| Downhill | 962.69 ± 256.21 | 47.66 ± 7.05 | 20.27 ± 3.06 |
| Uphill | 969.74 ± 263.94 | 49.01 ± 7.86 | 19.75 ± 2.95 |
| Perturbated | 899.30 ± 203.42 | 49.11 ± 8.52 | 18.45 ± 2.61 |

Table S2 *P*-values of the comparison of measurement conditions across all subjects during the baseline measurements (T0) of all study participants of the base of support (BOS), its length (BOS L) and its width (BOS W).

| Condition I | Condition II | BOS (cm^2^) | BOS L (cm) | BOS W (cm) |
| --- | --- | --- | --- | --- |
| Neutral | Downhill | 0.982 | 0.128 | 0.951 |
|  | Uphill | 0.993 | 0.501 | 1.000 |
|  | Perturbated | 0.329 | 0.589 | 0.703 |
| Downhill | Neutral | 0.982 | 0.128 | 0.951 |
|  | Uphill | 1.000 | 0.994 | 0.995 |
|  | Perturbated | 0.949 | 0.993 | 0.264 |
| Uphill | Neutral | 0.993 | 0.501 | 1.000 |
|  | Downhill | 1.000 | 0.994 | 0.995 |
|  | Perturbated | 0.925 | 1.000 | 0.618 |
| Perturbated | Neutral | 0.329 | 0.589 | 0.703 |
|  | Downhill | 0.949 | 0.993 | 0.264 |
|  | Uphill | 0.925 | 1.000 | 0.618 |

Table S3 Mean values ± standard deviation of the base of support (BOS), its length (BOS L) and its width (BOS W) during measurement timepoints baseline (T0) and post (T1) a 4-week break (group C) or training program (group T).

| Condition | Day | Groups | BOS (cm^2^) | BOS L (cm) | BOS W (cm) |
| --- | --- | --- | --- | --- | --- |
| Neutral | T0 | C | 1019.27 ± 154.92 | 51.83 ± 7.71 | 20.11 ± 2.22 |
|  |  | T | 1000.71 ± 175.01 | 53.64 ± 5.13 | 18.93 ± 2.56 |
|  | T1 | C | 1031.10 ± 225.11 | 52.77 ± 7.14 | 19.91 ± 2.11 |
|  |  | T | 1038.54 ± 275.38 | 54.59 ± 10.25 | 19.05 ± 1.44 |
| Downhill | T0 | C | 941.09 ± 213.15 | 47.90 ± 6.89 | 19.73 ± 2.52 |
|  |  | T | 984.29 ± 303.50 | 47.42 ± 7.57 | 20.81 ± 3.57 |
|  | T1 | C | 838.53 ± 154.52 | 47.18 ± 7.49 | 18.14 ± 2.67 |
|  |  | T | 905.04 ± 240.93 | 47.46 ± 7.38 | 19.11 ± 2.94 |
| Uphill | T0 | C | 994.55 ± 287.31 | 50.18 ± 9.31 | 19.67 ± 2.31 |
|  |  | T | 944.93 ± 251.29 | 47.84 ± 6.38 | 19.83 ± 3.61 |
|  | T1 | C | 964.16 ± 258.27 | 48.09 ± 7.98 | 20.08 ± 2.90 |
|  |  | T | 1015.45 ± 132.43 | 50.89 ± 5.06 | 20.03 ± 1.18 |
| Perturbated | T0 | C | 912.71 ± 209.53 | 48.12 ± 8.55 | 19.11 ± 2.34 |
|  |  | T | 885.90 ± 207.50 | 50.09 ± 8.84 | 17.78 ± 2.81 |
|  | T1 | C | 926.60 ± 162.49 | 48.78 ± 5.21 | 19.08 ± 1.94 |
|  |  | T | 880.29 ± 268.61 | 49.51 ± 7.58 | 17.71 ± 3.45 |

Table S4 *P*-values of the intra-group comparison of the base of support (BOS), its length (BOS L) and its width (BOS W) during measurement timepoints baseline (T0) and post (T1) a 4-week break (group 1) or training program (group 2).

| Group | Condition | BOS (cm^2^) | BOS L (cm) | BOS W(cm) |
| --- | --- | --- | --- | --- |
| C | Neutral | 0.894 | 0.789 | 0.831 |
|  | Downhill | 0.334 | 0.828 | 0.235 |
|  | Uphill | 0.779 | 0.531 | 0.729 |
|  | Perturbated | 0.886 | 0.849 | 0.980 |
| T | Neutral | 0.694 | 0.787 | 0.905 |
|  | Downhill | 0.455 | 0.990 | 0.207 |
|  | Uphill | 0.515 | 0.361 | 0.868 |
|  | Perturbated | 0.954 | 0.866 | 0.952 |

Table S5 *P*-values of the inter-group comparison of the base of support (BOS), its length (BOS L) and its width (BOS W) during measurement timepoints baseline (T0) and post (T1) a 4-week break (group 1) or training program (group 2).

| Day | Condition | BOS (cm^2^) | BOS L (cm) | BOS W (cm) |
| --- | --- | --- | --- | --- |
| T0 | Neutral | 0.805 | 0.544 | 0.286 |
|  | Downhill | 0.717 | 0.883 | 0.445 |
|  | Uphill | 0.686 | 0.522 | 0.907 |
|  | Perturbated | 0.777 | 0.619 | 0.264 |
| T1 | Neutral | 0.954 | 0.649 | 0.300 |
|  | Downhill | 0.472 | 0.935 | 0.446 |
|  | Uphill | 0.583 | 0.362 | 0.955 |
|  | Perturbated | 0.646 | 0.805 | 0.286 |


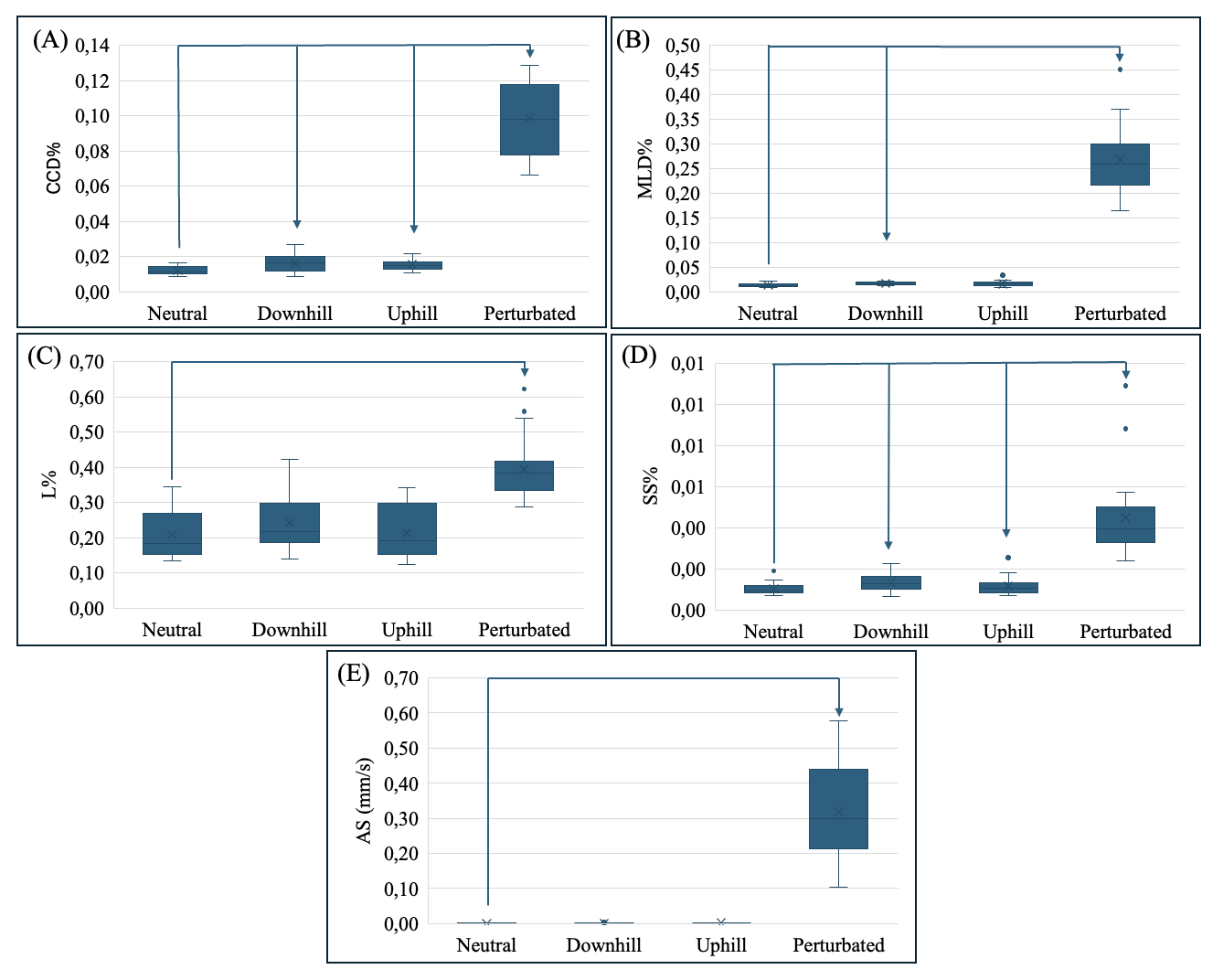


**Supplementary Figure 1** Pairwise comparison of the effect of measurement conditions during the first measurement (T0), including standing in a neutral position, standing downhill, standing uphill and perturbated standing on center of pressure (COP) parameters: (A) craniocaudal displacement (CCD%), (B) mediolateral displacement (MLD%), (C) statokinesiogram length (L%), (D) support surface (SS%) and (E) average speed (AS (mm/s)). Significant differences between conditions are marked with an arrow (p < 0.05).


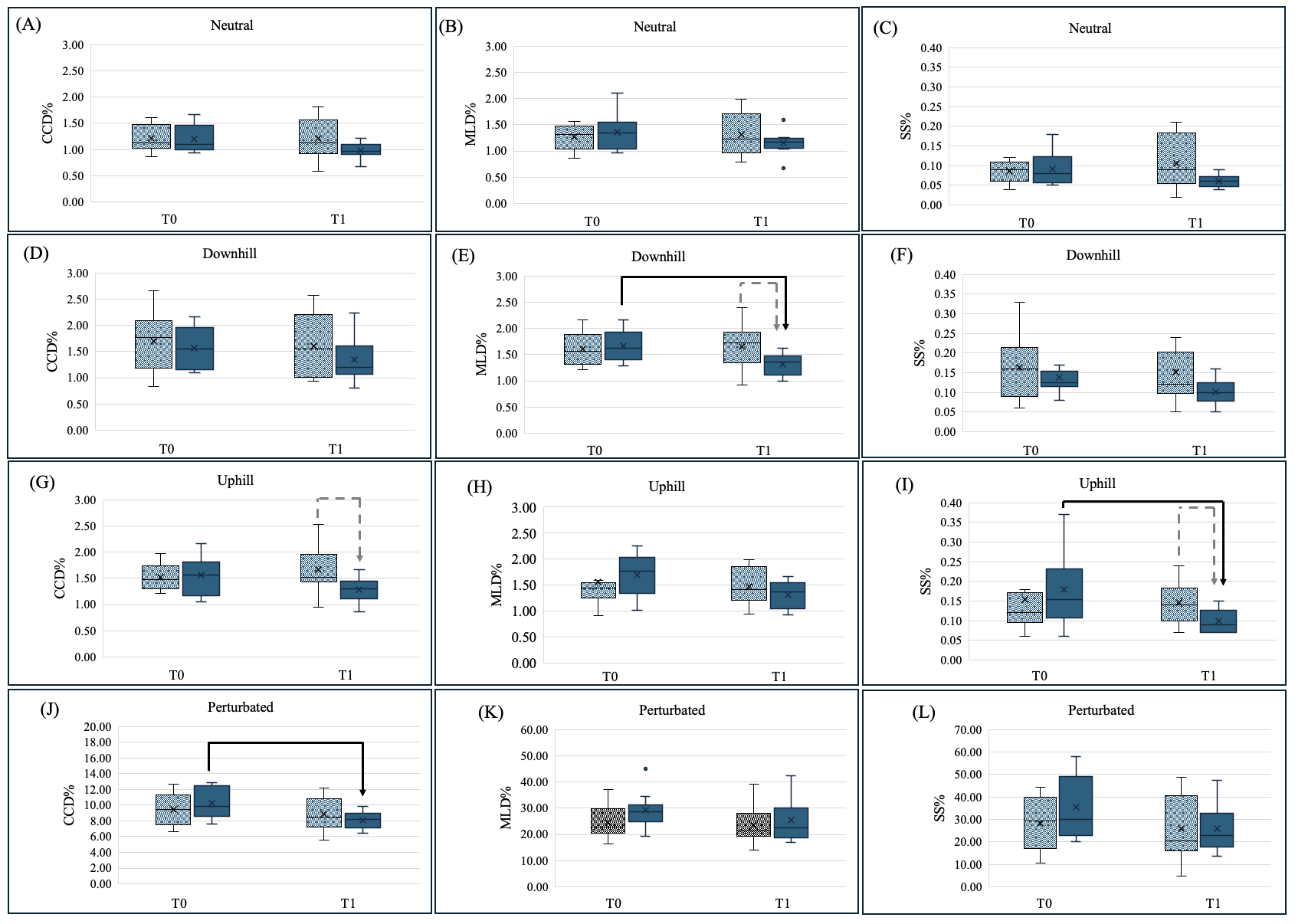


**Supplementary Figure 2** Pairwise comparison between groups and the effect of the measurement timepoints baseline (T0) and post (T1) for group C (control group, dotted-grey column) and group T (training program, solid blue column) for the conditions: (A-C) neutral standing, (D-F) uphill standing on a 20° slope, (G-I) downhill standing on a 20° slope, (J-L) perturbated standing. Center of pressure parameters include: craniocaudal displacement (CCD%), mediolateral displacement (MLD%) and support surface (SS%). Significant differences between measurement timepoints are marked with a black arrow and significant differences between groups are marked with a grey dotted arrow (p < 0.05).
